# Research on EV Crawler-Type Soil Sample Robot Using GNSS Information

**DOI:** 10.3390/s25030604

**Published:** 2025-01-21

**Authors:** Liangliang Yang, Chiaki Tomioka, Yohei Hoshino, Sota Kamata, Shunsuke Kikuchi

**Affiliations:** Laboratory of Bio-Mechatronics, Kitami Institute of Technology, Koentyo 165, Kitami Shi 090-8507, Hokkaido, Japan; m3245100375@std.kitami-it.ac.jp (C.T.); hoshinoy@mail.kitami-it.ac.jp (Y.H.); m3245100144@std.kitami-it.ac.jp (S.K.); m3235100182@std.kitami-it.ac.jp (S.K.)

**Keywords:** soil sampling, GNSS, IMU, auto-guidance, autonomous agricultural tractor, electric vehicle

## Abstract

In Japan, the decline in the number of agricultural workers and the aging of the workforce are problems, and there is a demand for more efficient and labor-saving work. Furthermore, in order to correct the rising price of fertilizer and the increasing burden on the environment caused by fertilizer, there is a demand for more efficient fertilization. Therefore, we aim to develop an electric soil sampling robot that can run autonomously using Global Navigation Satellite System (GNSS) information. GNSS and the Inertial Measurement Unit (IMU) are used as navigation sensors. The work machine is a crawler type that reduces soil compaction. In addition, a route map was generated in advance using the coordinate values of the field, with soil sampling positions set at 10 m intervals. In the experiment, the robot traveled along the route map and stopped automatically. The standard deviation of the standard deviation of lateral error was about 0.032 m, and the standard deviation of the interval between soil sampling positions was also less than 0.05 m. Therefore, it can be said that the accuracy is sufficient for soil sampling. It can also be said that even higher density sampling is possible by setting the intervals for soil sampling at finer intervals.

## 1. Introduction

The number of agricultural workers in Japan is on the decline, from about 1.75 million in 2015 to about 1.16 million in 2023. Additionally, the proportion of people aged 65 and over increased from 65% in 2015 to 71% in 2023 [1]. As a result, the workload and working hours per farmer are increasing, and labor-saving and efficiency improvements are required. In addition, the purchase price of fertilizers is currently soaring [2], and excess nutrients and deficiencies are becoming a problem in crops due to excess nutrients accumulated in the soil due to years of fertilization [3].

In previous research, various studies have been conducted to visualize and predict information such as soil nutrients and pH concentration through soil mapping, in order to carry out efficient fertilization. Gyawali et al. indicate that there are various techniques for soil sampling, and in order to obtain accurate sampling results, it is necessary to choose a sampling method that is appropriate for the effect on the target parameters [4]. Fan et al. have established a new wide-area DSM (digital soil mapping) method that adaptively takes into account the spatial distance to the sample using a widely applicable distance attenuation parameter value [5]. Zhang et al. proposed a multiple soil properties oriented representative sampling strategy (MPRS) as a method to design efficient sampling for multiple soil properties, taking into account influential environmental covariates for each soil property, and to accurately map multiple soil properties simultaneously [6]. In addition, Zhang et al. evaluated the effects of sampling density and depth in 3D soil mapping and found that lateral sampling density significantly affects the predictive accuracy of 3D mapping [7]. In order to commercialize digital soil mapping, Qu et al. conducted sampling at 12 different sampling densities and predicted the sand content of the soil. When the sampling density was 20% or less, the machine learning model was found to have an advantage [8]. Furthermore, as an example of the use of remote sensing and machine learning, Yuzugullu et al. were able to predict the amount of organic carbon when high-density sampling was performed using remote sensing and machine learning [9]. Safaee et al. evaluated the effects of sampling density, nutrient distribution, covariates, and modeling approach and demonstrated interactions with the effects of soil organic matter and cations on the accuracy of urea content predictions [10]. A three-dimensional predictive soil mapping approach was conducted by Hengl et al. to predict soil organic carbon (SOC) accumulation at high spatial resolution using a three-dimensional ensemble machine learning (3D-EML) framework [11]. Costa et al. showed that it is possible to accurately predict soil properties by using color parameters from the visible spectrum in a predictive model of soil components [12]. Furthermore, when Glavič-Cindro et al. examined the uncertainty of soil sampling using this type of machine learning prediction, they used both the conventional split sample method and an alternative method in which uncertainty is determined based on an evaluation of the standard deviation of the sample results. The results showed that the alternative method enabled reliable analysis with smaller sample amounts [13]. In addition, Yuan et al. developed a method for creating soil property maps at continental and national scales, enabling them to create large-scale soil property maps that are more accurate than maps created using conventional methods [14]. The generalized linear geostatistical model (GLGM) takes into account the non-stationarity of soil changes more than the commonly used regression clicking model, but it has not been studied much due to its drawback of complex calculations. M.-W. Zhang et al. used this model to map soil organic matter and compared it with other clicking models, and the results showed that more dense soil sampling is needed to explore and utilize the advantages of the GLGM [15]. In addition, as part of their research into machinery, Klopfenstein and Lussier Desbiens believed that soil sample collection robots were one of the causes of soil compaction, so they used a drone capable of high-speed flight to collect soil samples, achieving a high success rate on flat ground and reducing sampling time [16]. Numerous studies on automated driving also exist. Regarding algorithms, research is being conducted on autonomous driving systems using stereo cameras [17], autonomous driving systems using image recognition [18], autonomous driving systems using AI [19], and autonomous driving algorithms for agricultural assistance units [20]. Research is also being conducted on the GNSS, which is often used in autonomous driving. Nguyen and Cho evaluated the dynamic performance of GNSS positioning devices using low-cost positioning devices and legacy devices and showed that the performance of the low-cost positioning devices is accurate enough to be used as a positioning sensor for automatic guidance of agricultural vehicles [21]. Easterly et al. showed that the accuracy of automatic guidance systems using the GNSS is such that the error in the route increases significantly when the moving speed is fast compared to when it is slow, and that the error becomes infinitesimally small when a Real Time Kinematic (RTK)-level GNSS receiver is used [22]. Furthermore, in related research, Perez-Ruiz et al. developed a system that does not install an independent RTK-Global Positioning System (GPS) on the transplanter during transplanting work, but instead uses an RTK-GPS mounted on a tractor to map the location where the transplanter towed by it plants the crop, demonstrating that it is possible to create crop planting maps that are useful for plant care work such as fertilization and pesticide spraying during cultivation [23]. A simulator based on actual dynamic tests using an agricultural tractor was developed by Kaivosoja and Linkolehto to integrate the GNSS and dead reckoning systems to obtain sufficient positioning accuracy [24]. YIN et al. also developed an autonomous rice transplanter using the GNSS and IMU, and demonstrated that the lateral and longitudinal errors were less than 0.1 m and less than 5 degrees, respectively, relative to a straight path [25]. Kameyama et al. developed a soil sampling mechanism intended to be mounted on a small robot for use in paddy fields, and demonstrated that it is possible to sample soil using a robot equipped with the mechanism [26]. Noguchi et al. achieved an average lateral error of 0.035 m from the target path during work when using an autonomous agricultural vehicle robot, achieving driving accuracy far greater than that of humans [27].

Therefore, in this research, we develop a robot that automatically collects soil samples in the field. It determines soil sample collection points from GNSS information and connect the points to generate a route map. Then, while driving automatically, the vehicle corrects its position along the route based on GNSS and IMU values, moves to a point, and stops. In this study, the soil sample collection mechanism will be operated by a human.

## 2. Materials and Methods

### 2.1. Field of the Experiment

**Figure 1:**
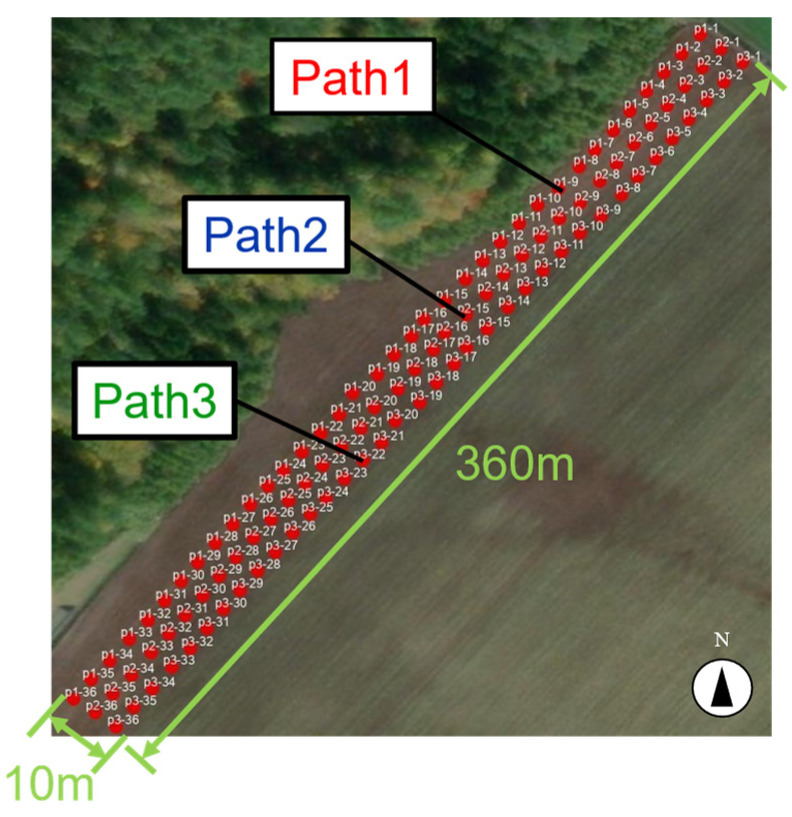
Points where soil samples were taken.

The experiment was conducted in an organic onion field at Yahagi Farm Ltd., located in Tsubetsu, Abashiri District, Hokkaido, Japan. The field measured approximately 360 m in length and 30 m in width. Soil sampling was performed in three rows, spaced 10 m apart from the edge along the short side of the field. Samples were collected every 10 m along each row, resulting in a total of 108 samples (Figure 1). The reason for setting the distance between sampling points at 10 m is that, when investigating the distribution of soil components, if the distance is too close, a large amount of similar data will be generated, and if the distance is too far, the component values will vary greatly between points. The value of 10 m was experimentally set to prevent extreme variation in component values due to distance. An aerial photograph of the organic onion field at Yahagi Farm is provided, with red dots marking the soil sampling points. The three rows are labeled as Path 1, Path 2, and Path 3, starting from the northwest side.

### 2.2. EV Crawler-Type Soil Sampling Robot

The development of the electric vehicle (EV) crawler-type soil sampling robot was undertaken in collaboration with Q-Hoe Co., Ltd., Ashoro Town, Ashoro District, Hokkaido, Japan, which was responsible for detailed design, manufacturing, and assembly. Additionally, the basic design of the soil sampling device was conducted by our team, while the detailed design, manufacturing, and assembly were outsourced to Sanyo Kogyo Co., Ltd., Hashinocho, Kitami City, Hokkaido, Japan.

**Figure 2:**
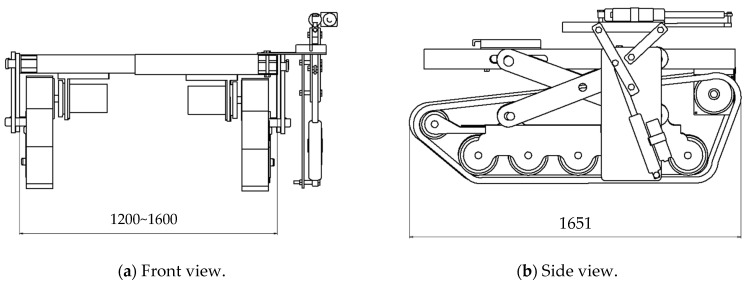
Drawing of the EV (electric vehicle) crawler-type soil sample robot used in the experiment.

As shown in Figure 2, the EV crawler-type soil sampling robot used in this experiment has a total length of approximately 1.6 m, a width that can be adjusted between 1.2 and 1.6 m, a height of approximately 0.88 m, and a weight of approximately 700 kg including the main body and soil sample collection mechanism. This robot is also a full crawler type, which means that it has a large contact area with the field, resulting in low ground pressure and allowing it to gently tread on the field as it moves, making it less likely to compact the field. Ground pressure is the load per unit area on the ground, and the average ground pressure for the total ground surface of the crawler is calculated by dividing the tractor’s weight by the total ground surface. Using this, the average ground pressure of the robot used in this study was calculated to be approximately 0.26 kgf/cm^2^, which is approximately 1/3 of the ground pressure of a typical tractor. From this, it can be said that a full crawler tractor is less likely to compact the field compared to a typical tractor, so a full crawler tractor was used in this study, taking the field into consideration.

The specifications of the EV crawler-type soil sampling robot are presented in Table 1. The system utilizes CAN (Controller Area Network) communication for data exchange between the motor drivers, which control the left and right motors, and the control PC. The robot operates with differential two-wheel control for navigation.

The specifications of the motor and motor driver are provided in Table 2 and Table 3, respectively. The battery specifications are summarized in Table 4.

**Figure 3:**
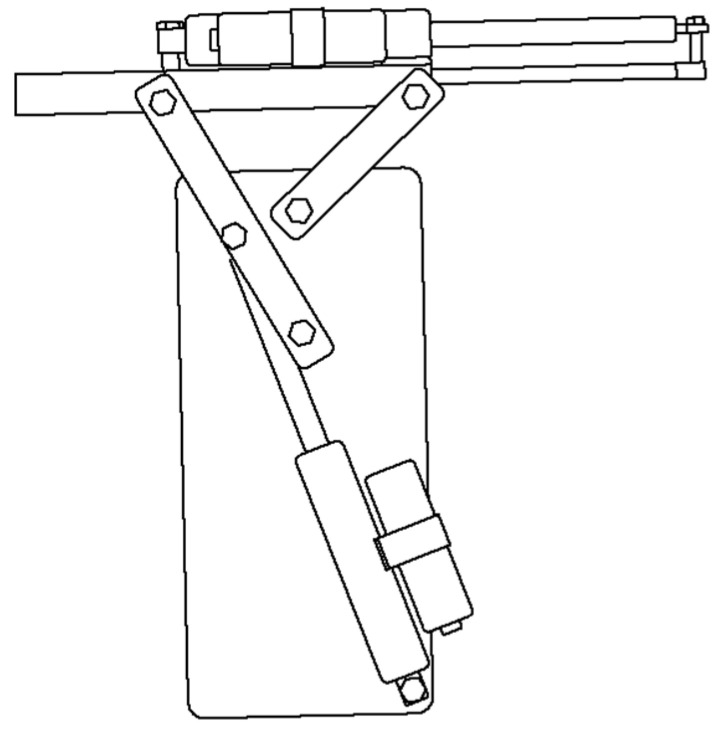
Soil sampling equipment.

**Figure 4:**
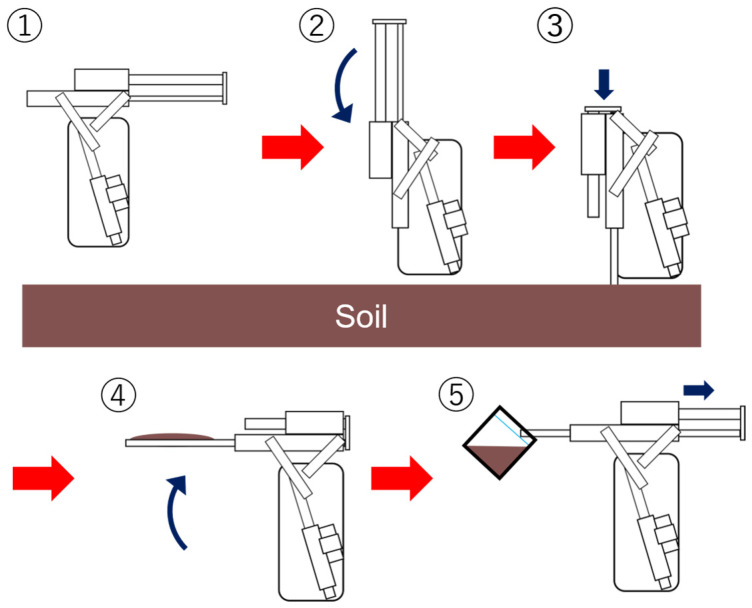
Soil sample collection procedure. The red arrows indicate the steps, and the blue arrows indicate the direction the mechanism moves.

The specifications of the electric hydraulic cylinder are outlined in Table 5. The electric hydraulic cylinder is connected to the soil sampling unit, and through a linkage mechanism, it moves the unit from a horizontal to a vertical position. Figure 3 shows an overall view of the unit. Its specific operation is shown in Figure 4. First, the unit in position ① is made perpendicular to the ground as shown in ②. Next, as shown in ③, the unit is inserted into the soil and a soil sample is taken. Next, as shown in ④, the unit is lifted up to a horizontal position with the tip still pointing into the ground. Finally, as shown in ⑤, the tip is returned to its original position and the soil sample is placed into a bag.

### 2.3. Equipment Used in Autonomous Driving System

The equipment used for autonomous driving are shown in Table 6. Navigation is achieved by mounting a laptop PC (Inspiron 14, Dell, Round Rock, TX, USA) on the EV crawler-type soil sampling robot and configuring the RTK-GNSS (DG-PRO1RW, BizStation Corp., Igawajo, Matsumoto City, Nagano Prefecture, Japan) using an Android Smartphone (FZ-N1, Panasonic, Higashi-Shinbashi, Minato Ward, Tokyo, Japan). For this experiment, the Ntrip Client was connected to the DOCOMO RTK system to receive the correction signal for the RTK-GNSS. The horizontal position accuracy of the RTK-GNSS at the fix condition was about 1 cm. An IMU (VN-100T-CR, VectorNav, Dallas, TX 75238, USA) was utilized to measure the heading of the vehicle and pitch and roll angle of the vehicle.

### 2.4. Autonomous Driving System

**Figure 5:**
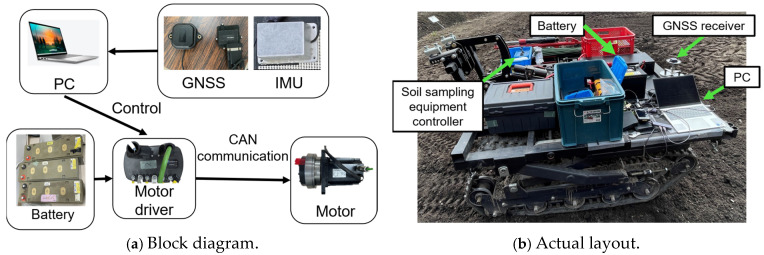
Block diagram of EV crawler-type soil sampling robot.

**Figure 6:**
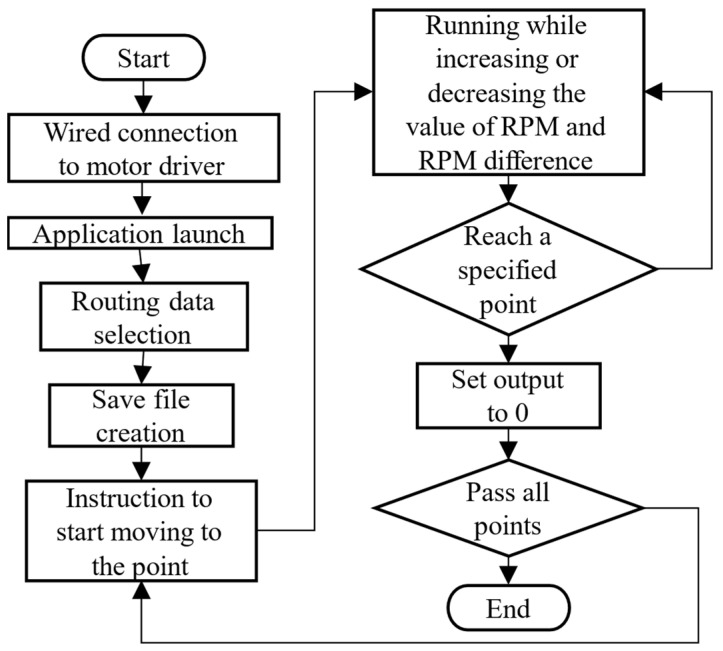
Flowchart of the program during automatic driving.

The block diagram and physical layout of the system are shown in Figure 5, while Figure 6 illustrates the flowchart of the autonomous driving system. Upon activation, the robot navigates using a pre-generated map of soil sampling points, along with real-time data from the GNSS and IMU. The robot continuously adjusts its position relative to the planned path and controls the motors via signals sent from the control PC to the motor drivers. This system enables the robot to autonomously move to the predefined stopping points on the map and halt at those locations. When further movement is required, the system sends a command to resume operation.

The calculation method for autonomous driving navigation begins with converting the GNSS-obtained coordinates into the ENU (East, North, Up) coordinate system. ENU coordinates have a certain point near the surface of the earth as the origin: the positive Z axis is in the direction of the zenith, the X axis is perpendicular to this in the east direction, and the Y axis is in the north direction. This allows you to calculate the distance, azimuth, and direction of an object from where you are standing. Equations (1) and (2) are then used to compute the speed differential between the left and right motors during the robot’s movement. The ENU coordinate system mentioned here is used to represent the horizontal and vertical planes of the position determined by the GPS, and the positioning error is expressed in this coordinate system.

(1) $$v_{R}=v_{T}\;\left[1+\min\left(2w,\;0\right)\right],$$

(2) $$v_{L}=v_{T}\left[1-\max\left(2w,\;0\right)\right],$$

where $v_{R}$ is the speed of the right motor, $v_{L}$ is the speed of the left motor, and $v_{T}$ is the target speed set for the motors when the vehicle is moving forward in a straight line, with a range of −3000 to 3000 rpm for this study. Furthermore, w is the vehicle turn rate and varies from −0.5 to 0.5.

In this experiment, the coordinate values obtained from the GNSS are transformed into a different coordinate system. Utilizing a World Geodetic System (WGS) 84-compliant ellipsoid that approximates the Earth’s shape, the transformation into an Earth-Centered, Earth-Fixed (ECEF) Cartesian coordinate system can be expressed by Equations (3) and (4).

(3) $$e=\frac{\sqrt{a^{2}-b^{2}}}{a},$$

(4) $$f=\frac{\left(a-b\right)}{a},$$

where *e* is the eccentricity, *f* is the oblateness, *a* is the semi-major axis (long radius), and *b* is the semi-minor axis (short radius).

Using the provided latitude φ, longitude λ, and the ellipsoid height h, the Cartesian coordinates, x, y, and z, are calculated according to Equations (5)–(7),

(5) $$x=\left(n+h\right)cos\varphi cos\lambda ,$$

(6) $$y=\left(n+h\right)cos\varphi sin\lambda ,$$

(7) $$z=\left[n\left(1-e^{2}\right)+h\right]sin\varphi ,$$

where *x*, *y*, and *z* are the values of the ENU coordinate system, respectively. Additionally, *n* and *e*^2^ are defined in Equations (8) and (9).

(8) $$n=\frac{a}{\sqrt{1-e^{2}{sin}^{2}\varphi }},$$

(9) $$e^{2}=2f-f^{2},$$

where *N* is the radius of curvature, while *e*^2^ is the square of the eccentricity.

Next, we transform the ECEF Cartesian coordinates into horizontal Cartesian coordinates. The position when the Cartesian coordinate system is rotated around the axis is expressed by the rotation matrix and the position before rotation as shown in Equation (10).

(10) Y=R · X,

where Y is the position after rotation, R is the rotation matrix, and X is the position before rotation. Then, if we define the matrices that rotate clockwise by θ around the x, y, and z axes, respectively, they can be expressed by Equations (11)–(13).

(11) $$R\left(x,\theta \right)=\left(\begin{array}{ccc} 1 & 0 & 0\\ 0 & \cos\theta & \sin\theta \\ 0 & -\sin\theta & \cos\theta \end{array}\right),$$

(12) $$R\left(y,\theta \right)=\left(\begin{array}{ccc} \cos\theta & 0 & -\sin\theta \\ 0 & 1 & 0\\ \sin\theta & 0 & \cos\theta \end{array}\right),,$$

(13) $$R\left(z,\theta \right)=\left(\begin{array}{ccc} \cos\theta & \cos\theta & 0\\ -\sin\theta & \sin\theta & 0\\ 0 & 0 & 1 \end{array}\right),,$$

Based on this, the positions E, N, U in the ENU coordinate system can be expressed by Equation (14) using the positions x, y, z in the ECEF coordinate system and the positions of the origin x_0_, y_0_, z_0_.

(14) $$\left(\begin{array}{c} E\\ N\\ U \end{array}\right)=R\left(z,90\right)\;R\left(y,90-\emptyset \right)\;R(z,\;\lambda )\left(\begin{array}{c} x-x_{0}\\ y-y_{0}\\ z-z_{0} \end{array}\right),$$

The above calculations are performed on coordinate data expressed in a geographic coordinate system that is preset as route data, and further analysis of autonomous navigation is performed using the ENU coordinate system values obtained by the calculations.

**Figure 7:**
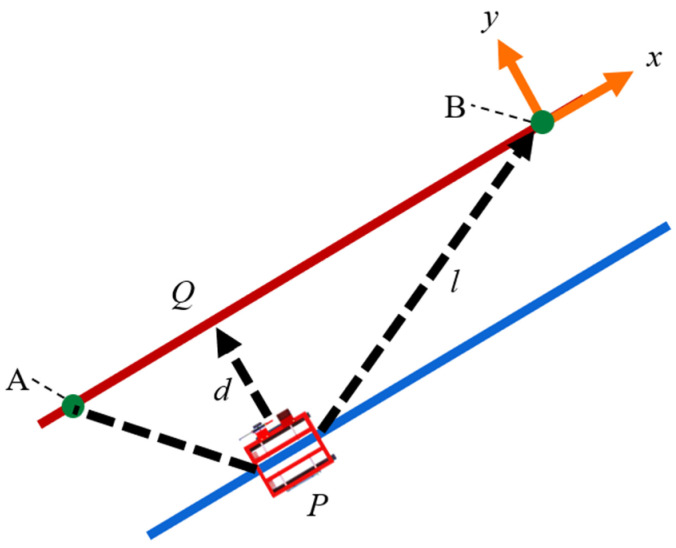
Calculating the navigated autonomous driving. Points and straight lines used when driving autonomously. Points A and B are certain points at the soil sample collection site in the field. The red line is the line between points A and B. The blue line is an auxiliary line obtained by moving the line AB parallel to the direction of the robot’s current location P. Q is the point obtained by moving P vertically on the line AB. x and y represent the x and y axes of the entire figure. d represents the distance between P and Q. l represents the distance between PB.

Next, we demonstrate the application of the calculated ENU coordinate system for navigating autonomous driving. Figure 7 presents a schematic diagram illustrating the calculation of the distance between points A and B. The coordinate values of points A and B are first converted to the ENU coordinate system, and navigation is subsequently performed based on these converted values.

As illustrated in Figure 7, let the current position of the EV crawler be designated as point P, the starting point as point A, and the target point as point B. When the robot moves from point A to point B, any deviation from the line connecting points A and B to the position of point P can be expressed by Equation (15),

(15) $$l=\left\|{\vec{v}}_{PB}\right\|,$$

where *l* is the length of the vector from point P to point B. The distance from point P, the current location of the robot, to the straight-line AB, which represents the target route, is expressed by Equations (16)–(18),

(16) $${\vec{v}}_{AP}={\vec{v}}_{P}-{\vec{v}}_{A},$$

(17) $${\vec{v}}_{AB}={\vec{v}}_{B}-{\vec{v}}_{A},$$

(18) $$d=\frac{{\vec{v}}_{AP}\times {\vec{v}}_{AB}}{\left\|{\vec{v}}_{AB}\right\|},$$

where *d* is the distance from the vehicle’s current position to the target path, ${\vec{v}}_{AP}$ is the vector from point A to point P, ${\vec{v}}_{AB}$ is the vector from point A to point B, × is the cross-product operator, and $\left\|{\vec{v}}_{AB}\right\|$ is the norm of ${\vec{v}}_{AB}$. The calculated value of d indicates the lateral error.

Additionally, the angle between the direction in which the robot is currently facing and the target point is expressed by Equation (19),

(19) $$\delta =atan2\left(dx,\;dy\right)*\;\frac{\pi }{180},$$

where δ is the angle between the direction in which the robot is currently facing and the target point. dx is the difference between the coordinates of the target and the current ENU coordinates, while dy is the difference between the n-coordinates of the target and the current ENU coordinates. Using this information, the heading error for the line segment AB is calculated by Equation (20),

(20) τ=α−δ,

where τ is the robot’s heading error relative to the line segment AB, while α is the current angle of the robot vehicle that is measured using the IMU and RTK-GNSS. After constraining τ to the range of −180 degrees to 180 degrees, the turning operation using PID control is expressed by Equation (21) using Equations (22) and (23),

(21) $$w_{1}=-\left(k_{P1}\emptyset +k_{I1}\sum_{i=-M}^{0}\emptyset_{i}+k_{D1}\dot{\emptyset }\right),$$

(22) $$w_{2}=-\left(k_{P2}d+k_{I2}\sum_{i=-M}^{0}d_{i}+k_{D2}\dot{d}\right),$$

(23) $$w=w_{1}+w_{2},$$

where w is the turning rate calculated using a PID control method based on the lateral distance error and the heading error between the vehicle’s direction and the target path. $w_{1}$ is the lateral error, while $w_{2}$ is the heading error. In this study, the PID parameters $k_{P1}$, $k_{I1}$, and $k_{D1}$ are set to 0.5, 0, and 2, respectively, $k_{P2}$, $k_{I2}$, and $k_{D2}$ are 0.5, 0, and 2, respectively. Additionally, M is set to 5.

### 2.5. Create the Route Map

A program developed in MATLAB (ver.9.14) was utilized to create the routes. Initially, the field designated for soil sample collection is visualized using Google Earth, allowing for the examination of the coordinate values at the start and end points of the route. These coordinates are represented in the LLH coordinate system (latitude, longitude, height, or geographic coordinate system). The ENU coordinate system for point B is first calculated with point A serving as the origin. Subsequently, the direction vector is determined using the calculated ENU coordinates of points A and B, as expressed by Equation (24).

(24) $${\vec{v}}_{AB}=B-A,$$

where ${\vec{v}}_{AB}$ represents the direction vector from point A to point B, with A and B denoting the ENU coordinates of each point. The next step involves normalizing this vector to create a unit vector. The norm of the vector ${\vec{v}}_{AB}$ is denoted as $\left\|{\vec{v}}_{AB}\right\|$, while $v_{ABx}$ and $v_{ABy}$ represent the x and y components of the vector ${\vec{v}}_{AB}$, respectively. The calculated $\left\|{\vec{v}}_{AB}\right\|$ is subsequently divided by 10 and rounded down to the nearest integer to determine the total number of points when the vector $\left\|{\vec{v}}_{AB}\right\|$ is segmented into 10 m intervals. The unit vector eAB is then computed by dividing the vector ${\vec{v}}_{AB}$ by norm $\left\|{\vec{v}}_{AB}\right\|$. Points are generated every 10 m in the direction of the calculated unit vector, and their coordinate values are obtained. By generating one row of points and simultaneously rotating the coordinate values, three rows of coordinate values are produced, which are designated as Path 1, Path 2, and Path 3, respectively. The transformation into the ENU coordinate system is as shown in Equation (3) through Equation (14). The specific programs used in MATLAB are shown below.
ptALLH = [A_lat,A_lon,A_hight]; ptBLLH = [B_lat,B_lon,B_hight]; ptBenu = lla2enu(ptBLLH,ptALLH,’flat’); ptA = [0, 0]’; ptB = [ptBenu(1),ptBenu(2)]’; vAB = ptB − ptA; nAB = norm(vAB); ptNums = floor(nAB/10); %10 is the space of point to point eAB = vAB/nAB; ex = eAB(1); ey = eAB(2); mapPoint = zeros(firstpointnumber,lastpointnumber); [m,n,z] = size (mapPoint); for i = 1:n newpt = [(i − 3) × 10, 0, 0]’; % 3 is total number of pathes mapPoint(:,i) = newpt; end formatted_points = sprintf(‘%.10f,%.10f,%.10f¥n’, mapPoint.’); rot = [ex, -ey,0; ey, ex,0; 0,0,1]; mapPoint = rot × mapPoint; mapPoint1 = mapPoint’; dxdydz = [0,9,0]’; dxdydz = rot × dxdydz; [m,n,z] = size(mapPoint1); dxdydzOffset = ones(m,n,z). × dxdydz’; mapPoint2 = mapPoint1 + dxdydzOffset; mapPoint3 = mapPoint2 + dxdydzOffset; llhpath1 = enu2lla(mapPoint1,ptALLH,’flat’); llhpath2 = enu2lla(mapPoint2,ptALLH,’flat’); llhpath3 = enu2lla(mapPoint3, ptALLH,’flat’); xyzENU = lla2enu([A_lat,A_lon,A_hight],[ B_lat,B_lon,B_hight],’flat’)

## 3. Results and Discussion

*Result of Soil Sampling Experiment*

**Figure 8:**
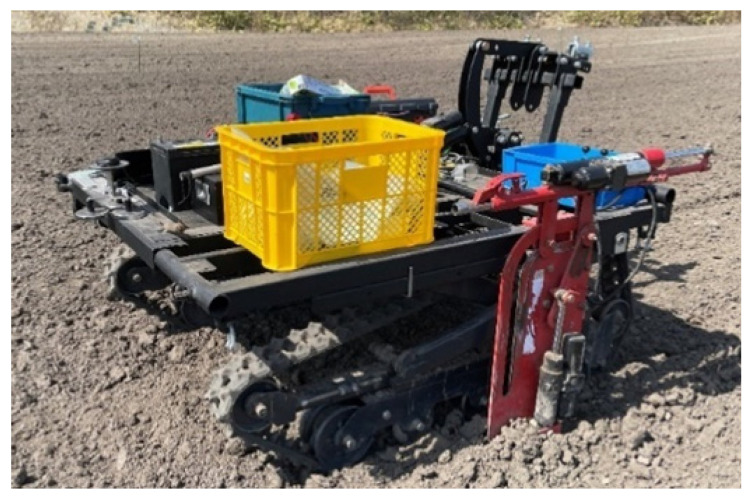
EV crawler-type soil sample robot.

The soil sampling experiment was conducted in April 2024, utilizing the implemented autonomous driving system. Figure 8 illustrates the EV crawler-type soil sampling robot employed in this experiment.

**Figure 9:**
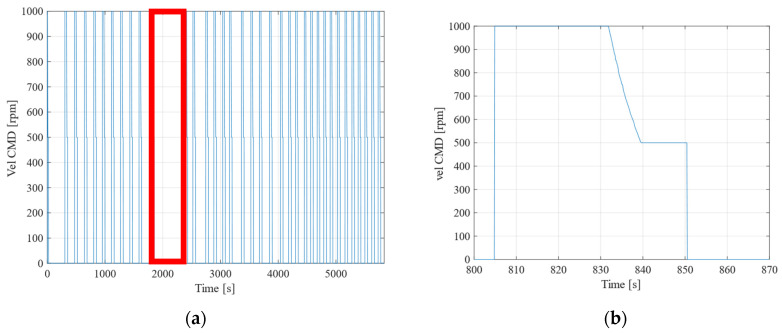
Changes in robot speed during automatic driving. (**a**) RPM value and time lapse. (**b**) RPM value specified by system and time elapsed (800 to 870 s).

Initially, Figure 9a presents a graph depicting the RPM values’ output during driving over time. Figure 9b provides an enlarged view of the interval from 800 to 870 s in Figure 9a. It is important to note that the space between approximately 1900 and 2400 s appears wider due to the robot being stationary during this period. From this graph, similar to the experiment detailed in Section 3, it can be observed that the RPM output is consistently at 1000 from the start of the run until the robot approaches the next point. Subsequently, the RPM decreases to 500, and eventually to 0 RPM.

**Figure 10:**
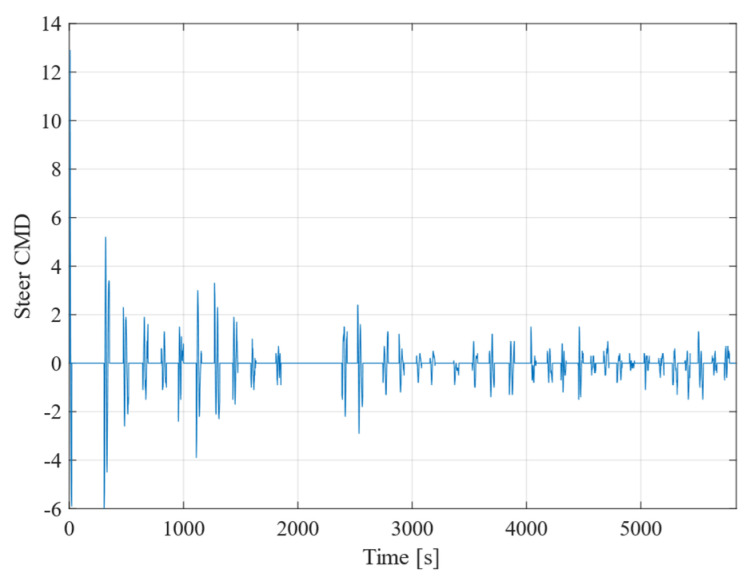
Change in value of difference between left and right RPM and time course.

Next, Figure 10 shows the change over time in the speed difference between the left and right motors. As the robot moves towards the target point, the steer command (Steer CMD) value increases in accordance with the robot’s turning motion. This value is substituted for w shown in Equations (1) and (2), and whether the robot will turn left or right is determined by whether the value is positive or negative. At first, the value is large because the deviation is large, but as time progresses the output value decreases and eventually stabilizes at or below 2. From this, we can see that the vehicle is able to travel without deviating significantly from the straight line connecting the target points.

**Figure 11:**
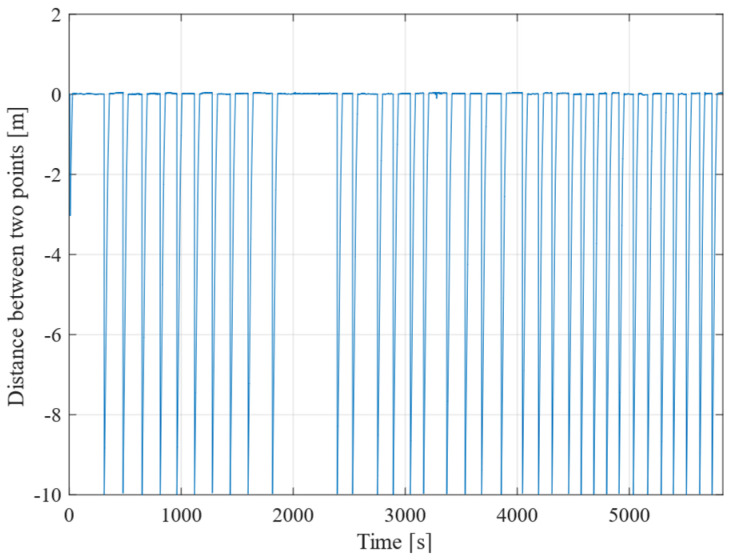
Distance between the previous point and the next point.

Next, Figure 11 illustrates the time variation in the distance between consecutive points. This figure demonstrates that the robot consistently reduces the distance from 10 m to 0 m each time it reaches a designated point, reflecting the changes in the target point. This pattern occurs in relation to the predetermined distance of 10 m set between the points.

**Figure 12:**
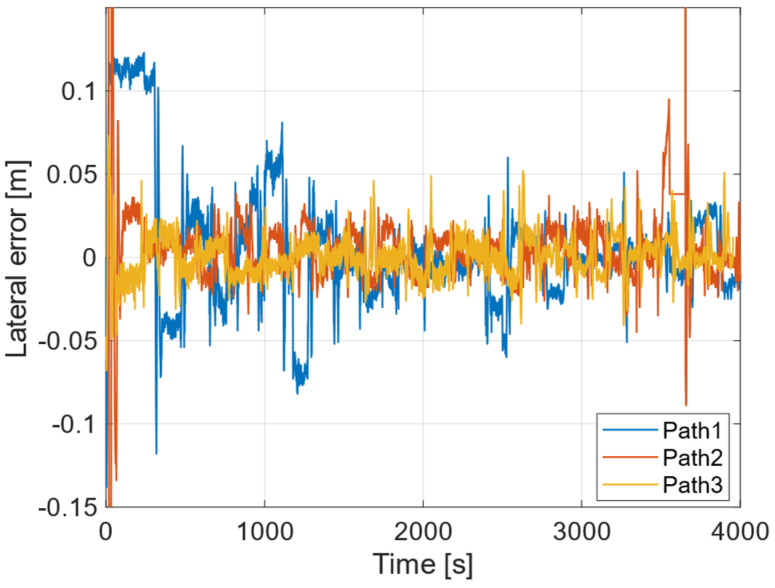
Lateral error of Path 1, Path 2, Path 3.

**Figure 13:**
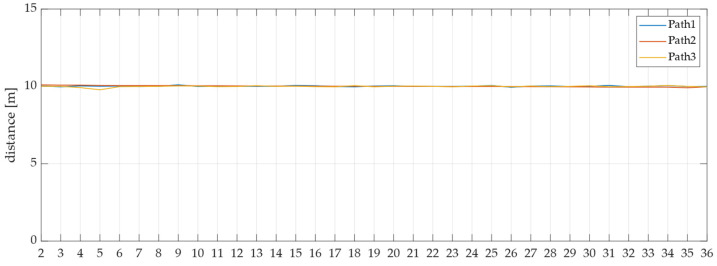
Distance between the previous point and the next point actually measured.

Finally, Figure 12 illustrates the changes in horizontal error over time for each row, from Path 1 to Path 3. These data are based on roll angle values obtained from an IMU. The corresponding standard deviations are presented in Table 7. Furthermore, Figure 13 depicts the distances measured between consecutive points for each path, from Path 1 to Path 3, with their respective standard deviations shown in Table 8. An examination of Table 7 reveals that all standard deviations are within 0.1 m, while Table 8 indicates that all standard deviations are within 0.05 m. These values are deemed sufficiently accurate for soil sample collection.

## 4. Conclusions

In this study, we investigated the autonomous operation of soil sample collection robots to reduce the workload associated with soil sampling and to streamline the collection process. Upon executing the completed program, the electric vehicle (EV) crawler-type soil collection robot successfully navigated and halted along the predefined route, based on coordinate values obtained in advance via the GNSS. The robot’s stopping positions accurately corresponded with the predetermined soil sampling point coordinates, demonstrating precise alignment between real-time coordinates and the robot’s position. Furthermore, the standard deviation of the left and right deviations from the route was minimal, approximately 0.032 m, indicating that the robot remained closely aligned with the designated path. The measured distances between actual stopping positions closely matched the target distance of 10 m. By utilizing a pre-constructed route map, the robot operated autonomously, enabling a single operator to efficiently collect soil samples.

The standard deviation of the lateral errors across the three rows was found to be within 0.1 m, while the standard deviation of the distances between each point was maintained within 0.05 m. Regarding the distance 10 m set in this experiment, we believe that if the distance is shortened, the lateral error and the error between sample collection points will be smaller, and if the distance is increased, the lateral error and the error between the sample collection points will be larger. Given that errors on the order of a few centimeters are not significant concerns during soil sample collection, these results indicate that the achieved accuracy is deemed sufficient for the intended purpose.

## Figures and Tables

**Table 1:** The specifications of the EV crawler-type soil sampling robot.

Specification	EV Crawler-Type Soil Sample Robot
Weight	700 kg
Number of motors	2
Motor output	1 kw × 2
Communication method	CAN communication
Driving type	Differential two wheels
Drive voltage	48 V
Battery	HS-DL48V100Ah

**Table 2:** The specifications of motor.

Specification	Motor
Model number	CHFM-5107P-SV-B-30
Ratio	30
Volts	25 V
Rating	3.15 N·m
r/min	3000
Output	1 kw

**Table 3:** The specifications of motor driver.

Specification	Motor Driver
Model number	AG120D4-2A000 HW2.2 FW 2.3.1
Input voltage	DC48 V–DC60 V
Output current	46 Arms–140 Arms (2s)
Communication method	CAN communication (CANopen)

**Table 4:** The specifications of battery.

Specification	Motor Driver
Model number	HS-DL48V100Ah
Nominal voltage	48 V
Capacity @ 20	300 min
Energy	4800 Wh

**Table 5:** The specifications of electric hydraulic cylinder.

Specification	Electric Hydraulic Cylinder
Model number	MMP5-B1B350AA
DC motor power	250 W
Relief valve setting pressure	7.1 MPa
Power supply	DC12 V
Cylinder size	φ40~φ20
Cylinder stroke	350 mm
Soil sampler model number	DIK-102A
Soil sampler diameter	φ30 mm

**Table 6:** The specifications of the devices used in the autonomous driving system.

Item	Model	Specification
Laptop PC	Inspiron14	Memory: 16 GB CPU: 12th Gen Intel(R) Core (TM) i7-1255U/1.70 GHz
Android terminal	FZ-N1	Memory: 4 GB CPU: Qualcomm^®^ SDM660 64bit
RTK-GNSS	DG-PRO1RWS	Horizontal position accuracy at RTK Fix: Horizontal: 0.02 m 2DRMS Vertical: 0.03 m 2DRMS
IMU	VN-100T-CR	Range (Heading/Yaw, Roll): ±180° Range (Pitch): ±90° Heading (Magnetic): 2.0° RMS Pitch/Roll (Dynamic): 1.0° RMS

**Table 7:** Standard deviation of lateral error.

	Path 1	Path 2	Path 3
Standard deviation [m]	0.032	0.076	0.012

**Table 8:** Standard deviation of distance between the previous point and the next point actually measured.

	Path 1	Path 2	Path 3
Standard deviation [m]	0.030	0.043	0.046

## Data Availability

Dataset will be made available on request to the authors.
